# A comparative study of incidence, mortality and disability adjusted life years (DALYs) for leading cancers in BRICS countries

**DOI:** 10.3332/ecancer.2024.1773

**Published:** 2024-09-19

**Authors:** Anita Nath, Ruchita Taneja, Yamini Saraswathi Thadi, Gokul Sarveswaran, Prashant Mathur

**Affiliations:** ICMR-National Centre for Disease Informatics and Research, Nirmal Bhawan, Kannamangala, Bengaluru 562110, Karnataka, India

**Keywords:** cancer, incidence, mortality, disability adjusted life years, BRICS

## Abstract

**Background:**

While cancer stands as a prominent global contributor to mortality, the BRICS countries, which contribute a considerable proportion of the world’s economy, also account for a substantial proportion of global cancer-related deaths. The study aims to compile data on the incidence, mortality and disability-adjusted life years (DALYs) of leading cancers in BRICS countries to assess any variations in these parameters.

**Methods:**

Indicators such as the age-standardised incidence rate (ASIR) and age-standardised mortality rate (ASMR) were abstracted from GLOBOCAN 2022. Global Burden of Disease 2019 provided an overview of DALYs. Additionally, ‘Cancer Tomorrow’ provided projections for future cancer rates up to 2050.

**Results:**

The findings revealed that Russia had the highest ASIR for both sexes. Among males, leading cancer sites included prostate, lung and colorectum, while India stood out with lip and oral cavity cancer as the primary site. Breast cancer dominated among females in all BRICS countries, except China, where lung cancer took precedence. South Africa recorded the highest ASMR for both sexes, with Russia leading among males and South Africa among females. Lung cancer has been the leading cause of death in all countries except India, where breast cancer leads. Trachea, bronchus and lung cancers contributed the most to DALYs, except in India, where breast cancer prevailed. South Africa and India are anticipated to have the highest increase in new cancer cases and deaths in future.

**Conclusion:**

Breast and lung cancers accounted for the highest incidence, mortality and DALYs in females and males, respectively. Although the BRICS countries anticipate sustained economic growth and have viable cancer control plans, it is essential to investigate cancer risk factors and health systems influencing cancer incidence and outcomes.

## Introduction

Cancer stands as a prominent global contributor to mortality, responsible for almost 10 million deaths in 2020, amounting to approximately one-sixth of all deaths, among which cancers of the lung, colon and rectum, liver, stomach and breast accounted for the highest number of deaths [1].

Based on 2019 estimates by the World Health Organisation (WHO), cancer emerges as the first or second leading cause of mortality under the age of 70 in 112 out of 183 countries while securing the third or fourth position in an additional 23 countries [2]. An estimated increase of 26.3%, 20.9% and 16.0% in cancer incidence, mortality and disability-adjusted life years (DALYs) has been observed between 2010 and 2019, with the highest increase in low and middle-income countries [3].

The occurrence of cancer and its outcomes is associated with socio-demographic, socio-cultural and behavioural risk factors and health systems that can differ among countries and may persist in specific geographic regions. The observed variations could also reflect disparities in the accessibility of cancer services, encompassing screening and treatment. In certain areas, this stems from a lack of infrastructure to deliver these services, while in others, the cost of cancer care, which comprises diagnosis, treatment and follow-up, renders them inaccessible to specific individuals. While densely populated and accounting for 42% of the world’s population, the BRICS nations, including Brazil, Russia, India, China and South Africa, are also experiencing swift economic changes and are expected to contribute towards a quarter of the global economy [4]. The economic changes in BRICS nations could have a considerable impact on cancer incidence, mortality and DALYs. Economic growth often enhances healthcare infrastructure, increases access to medical services and improves early cancer detection, potentially lowering mortality and DALYs. However, such growth can also exacerbate healthcare inequalities, with some groups benefiting more. Additionally, economic development can lead to lifestyle changes, such as increased consumption of tobacco, alcohol and processed foods and decreased physical activity, which may elevate cancer incidence rates.

Conversely, higher incomes and education can foster healthier lifestyles and preventive measures. Therefore, the overall effect of economic changes on cancer outcomes in BRICS nations is complex and multifaceted. The study aims to describe the incidence, mortality and DALYs of leading cancers in BRICS countries to assess any variations in these parameters. Detecting variations in cancer incidence, mortality and DALYs might signify diverse risk patterns that should inspire research into the causes, offer insights for allocating health resources and direct interventions to mitigate risks.

## Material and methods

The present study analysed relevant cancer-related indicators such as incidence, mortality and DALYs that were abstracted from global resources for the BRICS countries. Data on the age-standardised incidence rate (ASIR) and age-standardised mortality rate (ASMR) indicators were abstracted from GLOBOCAN 2022, a web-based repository. It comprises worldwide cancer statistics, including estimates for the incidence and mortality of 36 cancer types in 185 countries and comprehensive data for all combined cancer sites [5]. The Global Burden of Disease 2019, which gives a keen overview of mortality and disability on a global scale, considering factors such as countries, time, age and gender, provided data on DALYs [7]. The Global Cancer Observatory-Cancer Over Time showcases global patterns in cancer-specific incidence and mortality rates [8]. The incidence information relies on well-documented data collected by one or more population-based cancer registries at either subnational or national levels. Data on ASIR for two time periods for Brazil (1993 and 2012), China (1988 and 2012) and India (1983 and 2012) was abstracted from this resource. Data on the estimated number of new cancer cases and deaths in 2022 and 2045 was abstracted from the Global Cancer Observatory-Cancer Tomorrow. ‘Cancer Tomorrow’ offers a range of data visualisation tools for forecasting future cancer incidence and mortality rates in a specified country or region [9]. The predictions extend from the current estimates in 2022 to the year 2050, utilising projections for the absolute numbers, incidence, mortality and prevalence of 36 specific cancer types and overall cancer rates for both sexes-males and females and age groups 0–85+ years. These projections cover 185 countries or territories worldwide in 2022, categorised by sex and age group, as part of the comprehensive GLOBOCAN project. The data were abstracted and entered using Microsoft Excel and analysed using descriptive statistics.

### Ethics approval

The study was approved by the Institutional Ethics Committee NCDIR/IEC/3068/2023.

## Results

### ASIR of all cancers combined

**Figure 1.**
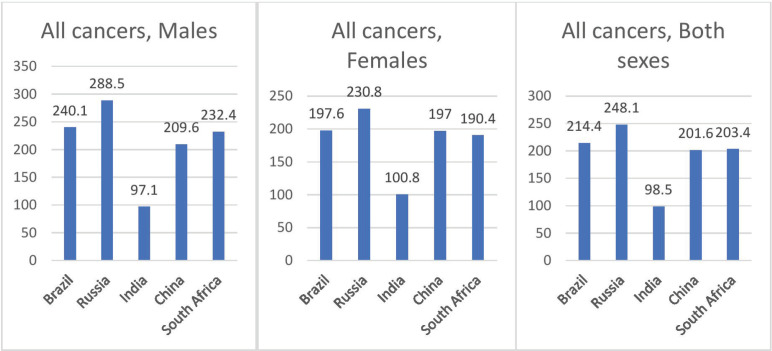
Age standardised incidence rate of all cancers in BRICS countries.

**Figure 2.**
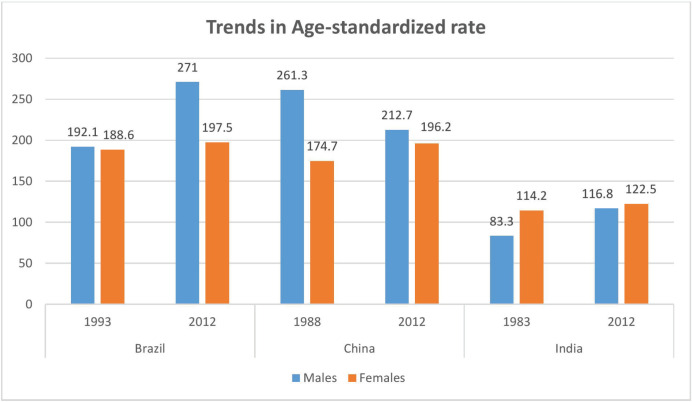
Comparison of standardised incidence rate at two different periods for Brazil, China and India.

The ASIR of all cancers combined among males, females and both sexes is shown in Figure 1. ASIR was highest in Russia in both the sexes combined (248.1 per 100,000), males (288.5 per 100,000) and females (230.8 per 100,000), followed by Brazil (214.4 per 100,000) and South Africa (203.4 per 100,000) for both sexes. Cervical cancer ranked as the second leading site of cancer among females in India and South Africa. A comparison of the ASIR of three countries at different periods, as shown in Figure 2, noted a decline in ASIR among cancer in males in China over nearly 25 years (261.3 per 100,000 in 1988 and 212.7 per 100,000 in 2012. There has been a remarkable increase in ASIR among males in Brazil (192.1 per 100,000 in 1993 and 271.0 per 100,000 in 2012). The rise in ASIR has also been noted in males in India and females in Brazil, India and China.

### Leading incident cancer sites

The commonest top three leading cancer sites among males comprised prostate, lung and colorectum. India is the only nation where cancer of the lip and oral cavity constituted the top leading site. Breast cancer accounted for the highest proportion of cases among females in all the countries except China, where lung cancer ranked as the first leading site. Cervical cancer ranked as the second leading site of cancer among females in India and South Africa.

### Age standardised mortality rate

**Figure 3.**
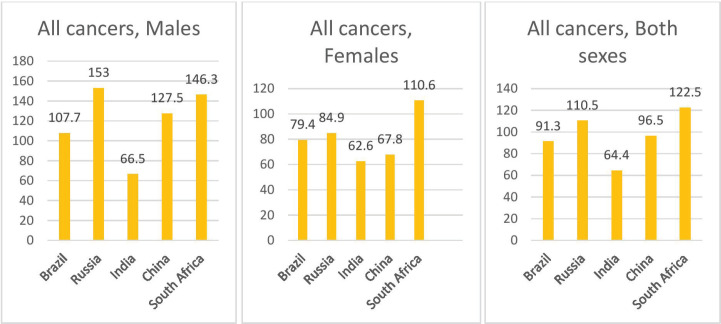
Age standardised mortality rate of all cancers in BRICS countries.

The highest ASMR due to all cancers was observed in South Africa for both sexes (122.5 per 100,000), in Russia for males (153.0 per 100,00) and among females in South Africa (110.6 per 100,000), as shown in Figure 3.

### Top five leading cancer sites accounting for deaths in both sexes

The leading cancer sites that contributed to cancer deaths are shown in Table 2. The highest number of deaths were due to lung cancer in all countries except India, where the leading cause of death was due to breast cancer.

### DALYs, leading cancers

Cancers of the trachea, bronchus and lung contributed to the highest DALYs in all the BRICS countries, except India, where breast cancer accounted for the highest DALYs 193.96 (150.3–246.24) as presented in Table 3.

### Estimated number of new cases and deaths from 2022 to 2045

**Figure 4.**
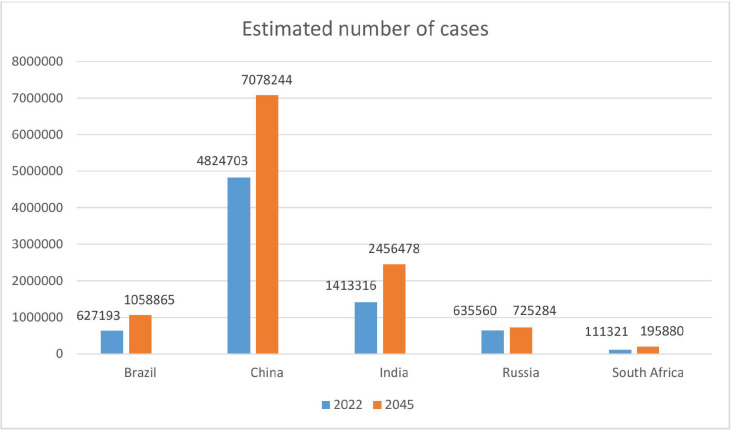
Estimated number of cases (0–85 years) from 2022 to 2045, in both sexes.

**Figure 5.**
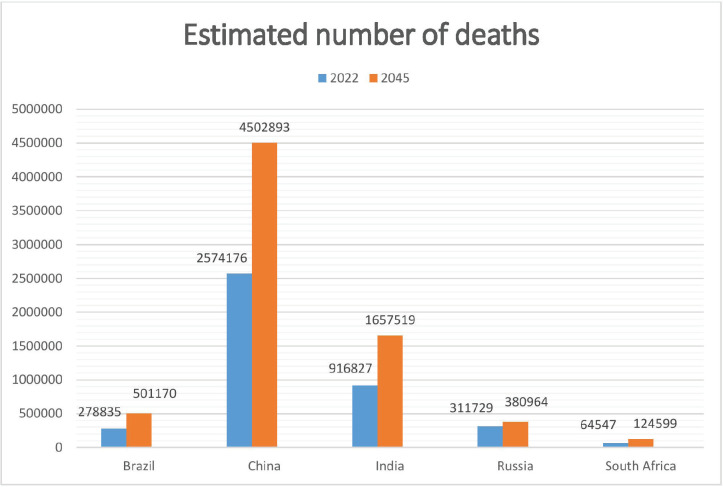
Estimated number of deaths (0–85 years) from 2022 to 2045 in both sexes.

The estimated number of new cases and deaths in 2022 and 2045 are represented in Figures 4 and 5, illustrating that South Africa and India are expected to witness the highest increase in new cancer cases and deaths as shown in Table 4.

### Data sharing statement

The data that support the findings of this study are available in https://www.who.int/data/gho/indicator-metadata-registry/imr-details/78; https://gco.iarc.fr/overtime; https://gco.iarc.fr/tomorrow;

## Discussion

This paper comprehensively analysed cancer incidence, mortality and DALYs in the BRICS nations. The findings also reflect upon the economic transition that is being experienced by these countries. The age-standardised cancer incidence ASIR was highest in Russia compared to other BRICS countries. The variations in the ASIR could be attributed to a nation’s development and socio-demographic profile. A strong correlation between the human development index (HDI) and cancer incidence has been observed; national incidence rates rise rapidly as HDI levels increase [10]. Russia has the highest HDI of 0.821 among all the five countries [11]. India recorded the lowest cancer incidence, which could be explained by the higher proportion (33%) of young population of less than 15 years [12]. On the contrary, Russia belongs to demographically old countries wherein the proportion of persons over 65 is 15% [13].

Compared to other nations, India was distinct in terms of reporting a high incidence of lip and oral cavity cancer. Oral cancer holds considerable public health significance in India. Earlier studies conducted in India have conveyed that the country has the highest occurrence of oral cancer, with smoked and smokeless tobacco forms having been identified as a leading cause and smokeless tobacco contributes to a higher risk of cancer in women [14]. From an economic perspective, low socioeconomic status can increase the risk of oral cancer, similar to the effect of lifestyle risk factors [15]. Breast cancer constituted one of the top cancer sites in terms of new case numbers in females in all five countries and DALYs in four countries, which socioeconomic progress can explain. A prior review indicated that BRICS countries accounted for 33.6% of new breast cancer cases worldwide and 36.9% of global breast cancer deaths. Additionally, these countries experienced a significant upward trend in breast cancer incidence [16]. Stomach cancers were among the top five leading sites among males in all BRICS countries except South Africa. China holds the highest number of stomach cancer cases, totalling 478,000, constituting 43.9% of the global incidence [17]. The high absolute numbers of cases of Kaposi sarcoma and Non-Hodgkins Lymphoma (NHL) in South Africa are reflective of the high incidence of HIV in the country [18]. Concerning changes in ASIR between the two-time points, it is interesting to note the rise in ASIR in both sexes in Brazil and India and females in China, except for Chinese males, where a decline was observed in 2012 compared to 1988. The ASIR in Chinese men has demonstrated a consistent and stable pattern over the preceding decades [19].

The age-standardised cancer mortality rate was highest in both genders combined and females in South Africa compared to other BRICS countries. Among all cancers, lung cancer accounted for the most increased mortality in China, Brazil, Russia and South Africa. The incidence and mortality rates of lung cancer are highest in economically developed countries where tobacco smoking was most prevalent several decades ago. However, these rates have mostly reached their peak and are now on the decline [20]. Cancer deaths significantly contribute to overall productivity loss. A study by Pearce *et al* [21] revealed that BRICS nations account for 42% of global cancer-related deaths. China experienced the highest total productivity loss at $28 billion, while South Africa incurred the highest cost per cancer death at $101,000. Our analysis also shows the sharp increase in cancer cases and deaths between 2022 and 2045 in India and South Africa. Sathishkumar *et al* [22] reported a 12.8% increase in the incidence of cancer cases in India in 2025 compared to 2020 and that cancer incidence is continuing to rise. The projected growth in cancer case numbers can be linked to the upsurge in life expectancy at birth, a trend observed across all BRICS countries from 2000 to 2022 [12].

The study underscores the significant role of BRICS countries in global cancer-related morbidity mortality and highlights the impact of economic changes on cancer incidence and outcomes in these nations. The progressive privatisation of healthcare has resulted in increased inequality, fragmentation of public health services and elevated levels of out-of-pocket spending – standard features observed in the BRICS countries. Our research highlights that policies promoting lifestyle changes to reduce cancer risk can positively impact the economies of BRICS countries. Integrating tobacco control, vaccination programs and cancer screening with access to proper treatment could substantially benefit public health and economic performance. The findings also highlight the need to strengthen cancer surveillance through cancer registration. While each BRICS country has established cancer registries, they face common challenges such as data completeness and resource limitations. Although the BRICS countries anticipate sustained economic growth and have viable cancer control plans, it is essential to investigate cancer risk factors and health systems influencing cancer incidence and outcomes. In light of these considerations, there is both a necessity and an opportunity for the BRICS nations to collaborate and learn from each other, thereby enhancing the overall progress of sustainable development.

## Conflicts of interest

None to declare.

## Funding

No funding was received to conduct this analysis.

## Figures and Tables

**Table 1.** Absolute percentage all cases in BRICS countries 2022 according to sex.

		Males			Females			Both sexes		
	Rank	Cancer site	No of cases	Percent	Cancer site	No of cases	Percent	Cancer site	No of cases	Percent
Brazil	1	Prostate	102,519	32.1%	Breast	94,728	30.8%	Prostate	102,519	16.3%
	2	Colorectum	30,164	9.4%	Colorectum	29,954	9.7%	Breast	94,728	15.1%
	3	Lung	24,804	7.8%	Thyroid	25,601	8.3%	Colorectum	60,118	9.6%
	4	Stomach	14,700	4.6%	Lung	19,409	6.3%	Lung	44,213	7.0%
	5	Bladder	12,090	3.8%	Cervix uteri	18,715	6.1%	Thyroid	31,385	5.0%
Russia	1	Lung	56,078	18.6%	Breast	78,839	23.5%	Colorectum	83,693	13.2%
	2	Prostate	52,712	17.5%	Colorectum	44,454	13.3%	Breast	79,455	12.5%
	3	Colorectum	39,239	13.0%	Corpus uteri	29,852	8.9%	Lung	70,362	11.1%
	4	Stomach	21,315	7.1%	Cervix uteri	18,369	5.5%	Prostate	52,712	8.3%
	5	Kidney	16,882	5.6%	stomach	17,568	5.3%	stomach	38,883	6.1%
India	1	Lip,oral cavity	107,812	15.6%	Breast	192,020	26.6%	Breast	192,020	13.6%
	2	Lung	58,970	8.5%	Cervix uteri	127,526	17.7%	Lip,oral cavity	143,759	10.2%
	3	Oesophagus	45,608	6.6%	Ovary	47,333	6.6%	Cervix uteri	127,526	9.0%
	4	Colorectum	43,360	6.3%	Lip,oral cavity	35,947	5.0%	Lung	81,748	5.8%
	5	Stomach	43,060	6.2%	Colorectum	26,678	3.7%	Oesophagus	70,637	5.0%
China	1	Lung	658,722	26.0%	Lung	401,862	17.5%	Lung	1,060,584	22.0%
	2	Colorectum	307,688	12.1%	Breast	357,161	15.6%	Colorectum	517,106	10.7%
	3	Liver	267,898	10.6%	Thyroid	341,211	14.9%	Thyroid	466,118	9.7%
	4	Stomach	246,550	9.7%	Colorectum	209,418	9.1%	Liver	367,657	7.6%
	5	Oesophagus	167,472	6.6%	Cervix uteri	150,659	6.6%	Breast	361,228	7.5%
South Africa	1	Prostate	12,903	24.6%	Breast	14,712	25.0%	Breast	14,990	13.5%
	2	Lung	6,053	11.6%	Cervix uteri	10,532	17.9%	Prostate	12,903	11.6%
	3	Colorectum	3,873	7.4%	Colorectum	3,465	5.9%	Cervix uteri	10,532	9.5%
	4	Kaposi sarcoma	2,529	4.8%	Lung	3,393	5.8%	Lung	9,446	8.5%
	5	NHL	1,959	3.7%	Corpus uteri	1,839	3.1%	colorectum	7,338	6.6%

Source: (GLOBOCAN 2022)

**Table 2.** Top five leading cancer sites accounting for deaths in both sexes in BRICS countries.

	Rank	Cancer site	No of deaths	%
Brazil	1	Lung	38,292	12.5
	2	Colorectum	28,884	9.2
	3	Breast	22,189	13.9
	4	Prostate	19,958	13.5
	5	Stomach	18,138	5.9
Russian Federation	1	Lung	51,887	18.6
	2	Colorectum	41,447	13.3
	3	Stomach	27,306	9.2
	4	Breast	22,259	13.7
	5	Pancreas	20,672	7.2
India	1	Breast	98,337	13.7
	2	Lip, oral cavity	79,979	5.6
	3	Cervix uteri	79,906	11.2
	4	Lung	75,031	5.3
	5	Oesophagus	66,410	4.7
China	1	Lung	733,291	26.7
	2	Liver	316,544	12.6
	3	Stomach	260,372	9.4
	4	Colorectum	240,010	8.6
	5	Oesophagus	187,467	6.7
South Africa	1	Lung	8,672	16.9%
	2	Cervix uteri	5,976	19.0%
	3	Prostate	5,411	30.7%
	4	Breast	5,232	17.0%
	5	Colorectum	4,591	8.9%

Source: GLOBOCAN 2022

**Table 3.** DALY of leading cancers, BRICS countries (all ages, 2019).

Leading cancers						
	Both sexes	DALY (95% UI)	Males	DALY (95% UI)	Females	DALY (95% UI)
India	Breast	193.96 (150.3–246.24)	Tracheal, bronchus and lung	222.39 (173–273.45)	Breast	392.5 (302.56–500.35)
	Stomach cancer	165.89 (141.42–192.82)	Lip & oral cavity	177.43 (135.89–225.13)	Cervical	229.44 (176.85–309.09)
	Tracheal, bronchus and lung	163.6 (134.59–193.52)	Other pharynx cancer	166.53 (128.5–207.16)	Stomach cancer	173.33 (133.61–215.14)
Brazil	Tracheal, bronchus and lung cancer	400.04 (380.54–418.51)	Tracheal, bronchus and lung cancer	475.65 (450.15–500.69)	Breast cancer	530.77 (499.15–565.67)
	Colon and rectum cancer	297.57 (282.2–310.35)	Prostate cancer	390.58 (340–572.44)	Tracheal, bronchus, and lung cancer	327.89 (305.98–349.39)
	Breast cancer	276.03 (259.9–293.88)	Stomach cancer	335.79 (318.08–353.83)	Cervical cancer	314.27 (292.44–364.67)
China	Tracheal, bronchus and lung cancer	1,204.25 (1,008.22–1,422.39)	Tracheal, bronchus and lung cancer	1,651.13 (1,293.33–2,062.9)	Tracheal, bronchus, and lung cancer	739.87 (593.36–910.81)
	Stomach cancer	690.76 (575.93–817.86)	Stomach cancer	984.52 (777.23–1,212.18)	Breast cancer	412.49 (333.13–503.71)
	Colon and rectum cancer	449.6 (384.03–520.88)	Esophageal cancer	637.72 (490.1–801.63)	Stomach cancer	385.5 (309.99–477.96)
Russia	Tracheal, bronchus and lung cancer	917.16 (777.03–1,076.95)	Tracheal, bronchus and lung cancer	1,592.89 (1,303.82–1,916)	Breast cancer	811.57 (673.13–977.36)
	Colon and rectum cancer	640.55 (560.8–728.88)	Colon and rectum cancer	668.14 (553.71–798.16)	Colon and rectum cancer	616.51 (515.16–729.84)
	Stomach cancer	494.67 (430.96–569.18)	Stomach cancer	636.09 (524.05–763.95)	Stomach cancer	371.46 (307.45–444.03)
South Africa	Tracheal, bronchus and lung cancer	398.64 (355.63–460.86)	Tracheal, bronchus and lung cancer	575.77 (502.75–679.32)	Breast cancer	509.34 (442.45–586.55)
	Breast cancer	264.9 (230.47–304.65)	Prostate cancer	384.84 (310.18–451.51)	Cervical cancer	497.29 (404.88–592.12)
	Cervical cancer	253.71 (206.57–302.1)	Esophageal cancer	291.01 (251.66–386.1)	Tracheal, bronchus, and lung cancer	228.6 (198.49–263.29)

Source: Global Burden of Disease (GBD) 2019

**Table 4.** Percentage change in number of new cancer cases and deaths in 2022 and 2045.

Number of new cancer cases			
	2022	2045	% Change in number
Brazil	627,193	1,058,865	68.80%
China	4,824,703	7,078,244	46.70%
India	1,413,316	2,456,478	73.80%
Russia	635,560	725,284	14.10%
South Africa	111,321	195,880	76.00%
**Number of cancer related deaths**			
Brazil	278,835	501,170	+79.7%
China	2,574,176	4,502,893	+74.9%
India	916,827	1,657,519	+80.8%
Russia	311,729	380964	+ 22.2%
South Africa	64,547	124,,599	+93.0%

Source: Global Cancer Observatory-Cancer Tomorrow

## Supplementary text

### Definitions

- ASIR: The ASIR is a consolidated rate that reflects what would have been observed in a population with the age distribution of a specified reference population, commonly referred to as the standard population, based on the schedule of age-specific rates [6].
- ASMR: The ASMR is a calculated average of age-specific mortality rates per 100,000 individuals, utilising weights determined by the proportions of individuals in the respective age groups of the WHO standard population [7].
- DALYs attributed to a disease or health condition result from the combination of Years of Life Lost to premature mortality (YLLs) and Years Lived with Disability (YLDs) caused by a said disease or health condition within a population.
